# The role of KLF4 in human primordial germ cell development

**DOI:** 10.1098/rsob.240214

**Published:** 2025-01-22

**Authors:** Sun-Min Lee, Merrick Pierson Smela, M. Azim Surani

**Affiliations:** ^1^ Gurdon Institute, Tennis Court Road, University of Cambridge, Cambridge CB2 1QN, UK; ^2^ Department of Physics, Konkuk University, Seoul 05029, Republic of Korea; ^3^ Wyss Institute, Harvard University, Boston, MA 02215, USA; ^4^ Physiology, Development and Neuroscience Department, University of Cambridge, Cambridge CB2 3EL, UK

**Keywords:** KLF4, primordial germ cells, stem cells, pluripotency factors

## Abstract

Primordial germ cells (PGCs) are the founder cells that develop into mature gametes. PGCs emerge during weeks 2–3 of human embryo development. Pluripotency genes are reactivated during PGC specification, including Krüppel-like factor KLF4, but its precise role in PGC development is unclear. Here, we investigated the role of KLF4 in PGC development using our *in vitro* model for human PGC-like cells (hPGCLCs). We demonstrate that the depletion of KLF4 reduces the efficiency of hPGCLC specification, resulting in hPGCLCs with an aberrant transcriptome. Cut-and-run and transcriptomic analyses reveal that KLF4 represses somatic markers involved in neuronal and endodermal differentiation while promoting the expression of genes associated with PGC specification, such as *PAX5*, and epigenetic regulators, including *DNMT3L* and *REST*. KLF4 targets in hPGCLCs showed significant co-enrichment of motifs for SP and STAT factors, which are known to regulate cell cycle and migration genes. KLF4 contributes to human PGC development by activating genes involved in PGC specification and cell cycle regulation, while repressing somatic genes to maintain PGC identity.

**Figure:**
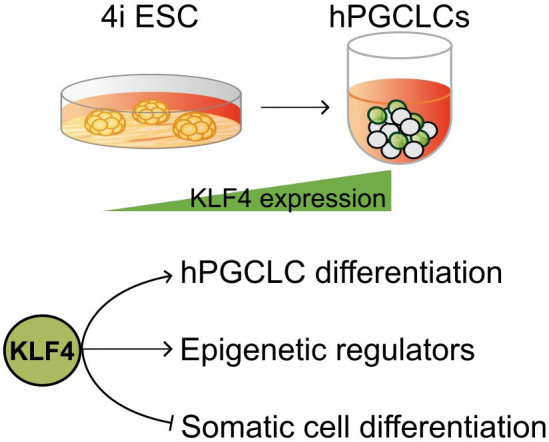

## 1. Introduction

Human primordial germ cells (hPGCs), the precursors of sperm and eggs, emerge during weeks 2−3 within gastrulating embryos [1]. The migration of hPGCs into the developing gonads typically occurs around weeks 5−6, where they undergo further differentiation through sex-specific programmes. This intricate process entails significant epigenetic reprogramming, leading to histone modifications and DNA demethylation, including the erasure of genomic imprints and the reactivation of the inactive X-chromosome [2]. To develop into functional gametes, primordial germ cells (PGCs) must undergo a specialized developmental pathway, involving the repression of somatic genes and activation of factors specific to pluripotency and germ cells [3,4].

The bone morphogenetic protein (BMP)–SMAD and WNT3–β-catenin signalling pathways are crucial for initiating the gene regulatory network responsible for PGC specification in both human and mouse embryos [1]. In humans, WNT signalling activates *EOMES*, leading to the expression of *SOX17*, a critical specifier of hPGCs [5]. Whereas in mice, WNT signalling induces *T* and *MIXL1*, which is required for the expression of *PRDM1* and *PRDM14*, the key mouse PGC (mPGCs) specifiers [6,7]. Notably, SOX17 is critical for hPGC specification but not for mPGCs [8]. PRDM1 represses somatic genes, including endodermal and mesodermal genes in both mouse and human PGCs [3,7].

The re-expression of pluripotency genes is a characteristic feature during PGC development. Expression of *POU5F1* (*OCT4*) is vital for the survival of PGC and for cooperation with SOX17 to induce germline-specific genes [9,10]. TFAP2C is a key regulator of human ground-state naive pluripotency, resembling the pre-implantation epiblast. Furthermore, TFAP2C also plays a crucial role in activating enhancers to establish a human germline programme, working alongside SOX17 [11,12]. By contrast, Krüppel-like factor 4 (KLF4) is actively repressed during the establishment of the mouse germline, where neither KLF4 nor SOX17 has a role in the germline [2,13]. Other genes associated with naive pluripotency, such as *NANOG*, *SOX15*, *KLF4* and *TBX3* are induced during hPGC specification [3,4].

Pluripotency factors are known to be expressed similarly in the pre-implantation epiblast and PGCs, sharing many characteristics. It is not well understood how their roles differ between these cell types. KLF4, a zinc finger transcription factor, is one of the ‘Yamanaka’ factors used for induced pluripotency in somatic cells and shows abundant expression during the transition from primed to naive pluripotency [14,15]. The naive pluripotent state is modelled *in vitro* by culturing human embryonic stem cells (ESCs) in media such as t2i/L/Go (t2iLGo) or 4-5iLA/F (5iLAF) [16–18]. KLF4 is also enriched in naive-specific accessible genomic sites in human naive ESCs. It plays a regulatory role in activating transposable elements, which can act as enhancers to drive the expression of nearby genes [19]. Depletion of KLF4 leads to the collapse of the naive pluripotent state [16]. However, the role of KLF4 in developing hPGCs is not well understood.

In this study, we report on the regulatory function of KLF4 in human PGC development. We demonstrate that KLF4 is essential for optimal germ cell fate, as its loss of function diminishes the efficiency of specification and leads to abnormal human PGC-like cells (hPGCLCs) transcriptome. We also identified target genes of KLF4 in hPGCLCs, revealing distinctive binding patterns compared with those in naive ESCs. Our study shows that KLF4 plays a significant role in the differentiation and maintenance of hPGCLCs by regulating gene activation and repression.

## 2. Results

### 2.1. KLF4 is upregulated during the induction of hPGCLCs *in vitro* and is expressed *in vivo* in hPGCs

**Figure 1.**
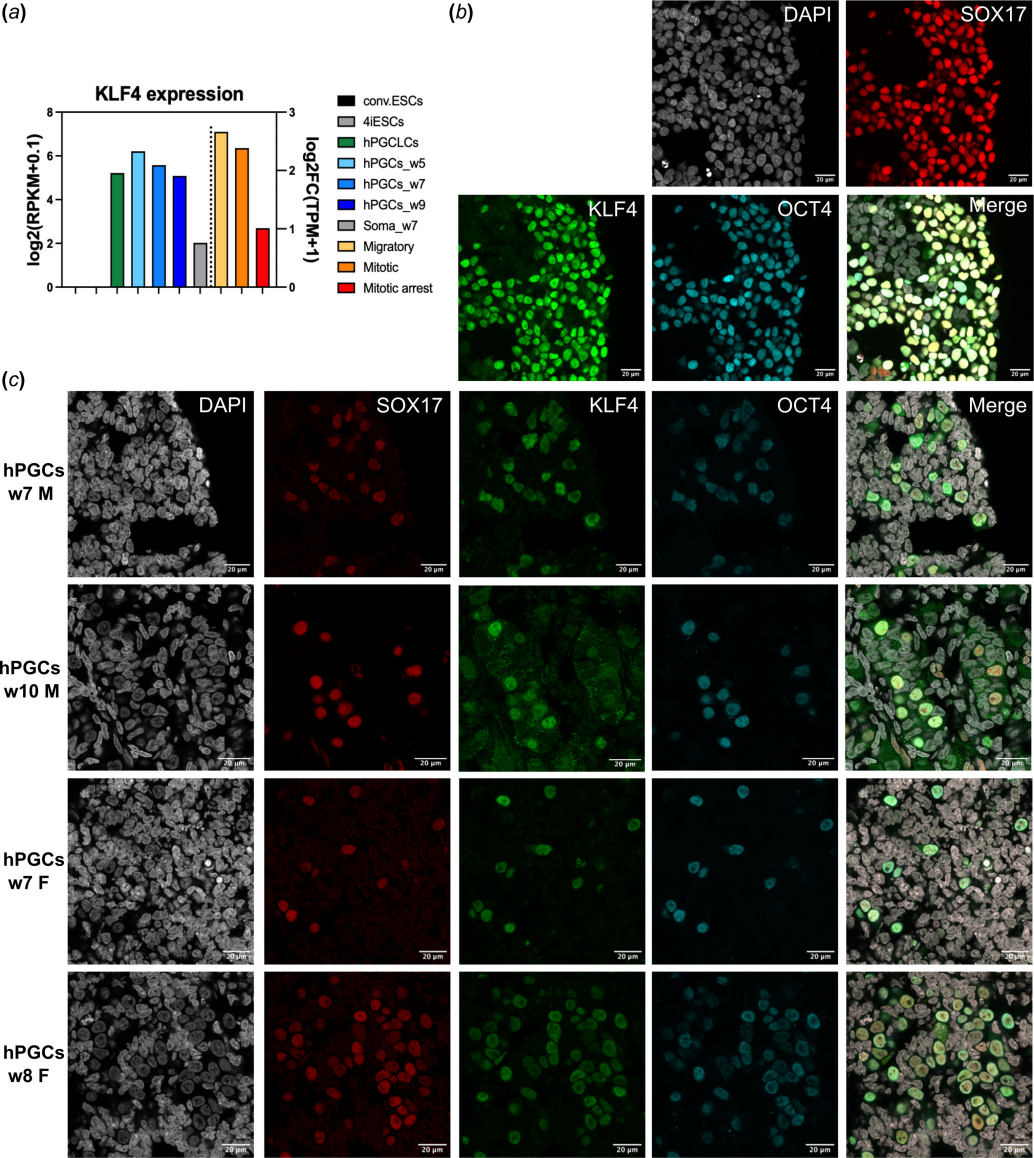
KLF4 expression is observed during both hPGCLC specification and *in vivo* hPGC development. (*a*) RNA-seq data of *in vitro* cells (conventional ESC, 4iESC, day 4 hPGCLCs) and *in vivo* hPGCs (week 5−9 hPGCs and soma, migratory, mitotic and mitotic arrest PGCs). Vertical dashed lines separate the two datasets based on their different vertical scales. (*b*) Immunofluorescence analysis showing the expression of KLF4 protein in day 4 hPGCLCs that co-express SOX17 and OCT4. Scale bars show 20 μm. (*c*) Immunofluorescence analysis revealed the expression of KLF4 protein in prenatal ovaries (weeks 7 and 8) and testes (weeks 7 and 10). hPGCs, identified by the expression of SOX17 and OCT4, exhibited KLF4 expression, while the surrounding soma did not show KLF4 expression.

We examined the levels of KLF4 expression using bulk RNA-seq data from different *in vitro* cell types, and *in vivo* hPGCs identified as tissue-specific alkaline phosphatase (TNAP) + KIT + cells collected at weeks 5, 7 and 9 of human fetal development [2]. We also used single-cell RNA sequencing to classify hPGCs into migratory, mitotic and mitotic arrest states [20]. We did not detect KLF4 expression in conventional human ESCs, and 4iESCs that are competent for hPGCLCs fate after culture in the presence of four inhibitors: GSK3, MEK, p38 and JNK, along with FGF2, TGFβ and LIF [3]. The 4iESCs differentiated into hPGCLCs in response to BMP2. KLF4 expression is upregulated in hPGCLCs compared with 4iESCs (figure 1*a*). We also observed sustained expression of KLF4 in bulk hPGCs from weeks 5 to 9 compared with somatic cells. Notably, KLF4 expression was observed during the migratory and mitotic stages of *in vivo* hPGCs, decreasing during the mitotic arrest (male) stage starting from week 9 onwards, coinciding with germline commitment and the downregulation of pluripotency genes (figure 1*a*). *In vitro*, hPGCLCs represented the early specification stage, exhibiting detectable KLF4 expression. We also confirmed that KLF4 expression is maintained from the migratory stage to the gonadal stage until just before sex-specific germline commitment, using hPGC transcriptome analysis.

For the experiments *in vitro*, we initiated the differentiation of hPGCLCs from 4iESCs and conducted immunofluorescence analysis of aggregates on day 4 to evaluate KLF4 protein expression in the newly induced hPGCLCs. Our results demonstrate strong expression of KLF4 in hPGCLCs (figure 1*b*), which were identified by co-expression of SOX17 and OCT4. Additionally, we examined the expression of KLF4 protein in the nuclei of *in vivo* hPGCs in both male and female gonads, specifically at weeks 7 and 10 for males, and weeks 7 and 8 for females (figure 1*c*). Our analysis showed KLF4 expression in hPGCs but not in the surrounding somatic cells.

### 2.2. Inducible degradation of KLF4 reduces hPGCLC specification

**Figure 2.**
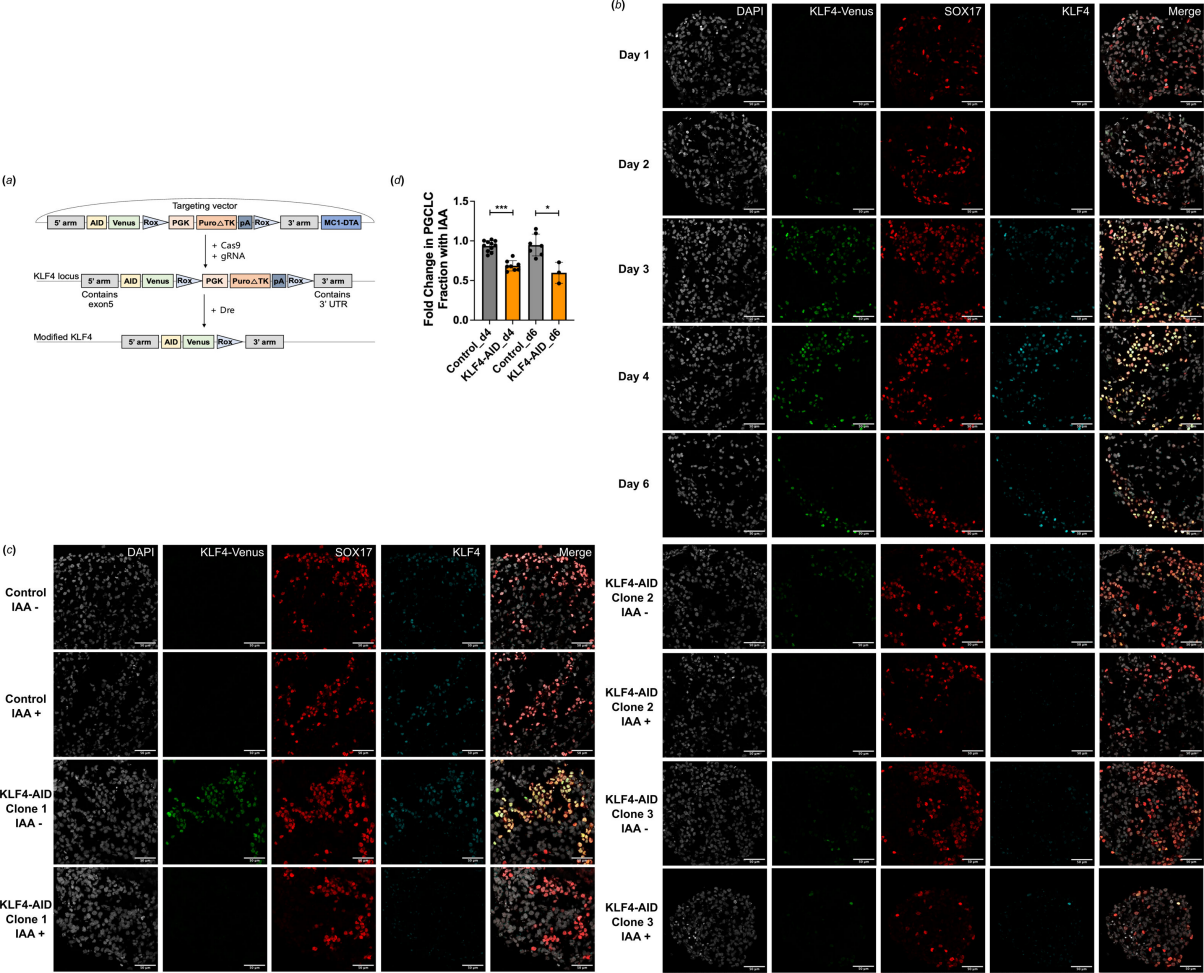
KLF4 depletion using AID degrons reduces hPGCLCs specification efficiency. (*a*) Scheme of CRISPR/Cas9-mediated KLF4 locus targeting to generate AID-Venus reporter versions. 5′ and 3′ arms-homology sequences, AID-auxin inducible degron, Venus—fluorescent gene, Rox-sequences for site-specific recombination recognized by the Dre enzyme, PGK-Puro—puromycin resistance gene under the control of PGK promoter, ΔTK-truncated thymidine kinase gene, MC1-DTA-diphtheria toxin fragment A gene under the control of MC1 promoter. (*b*) Time-course immunofluorescence showing KLF4 expression in embryoid body (EB) sections throughout hPGCLCs specification from the KLF4-AID-Venus fusion reporter cell line. Representative images for days 1, 2, 3, 4 and 6 of EB are shown. hPGCLCs were marked by SOX17. Scale bar is 20 μm. (*c*) Immunofluorescence images showing KLF4-Venus, SOX17 and KLF4 expression and KLF4-AID-Venus depletion upon IAA treatment in hPGCLCs. Nuclei were counterstained by DAPI and hPGCLCs were marked by SOX17. Scale bars are 20 μm. (*d*) The hPGCLC induction efficiency upon depletion of KLF4. NANOS3-tdTomato +TNAP + hPGCLCs yield in KLF4-AID hPGCLCs treated with IAA starting at day 0.

To investigate the role of KLF4 in hPGC development using the hPGCLC model, we generated an inducible depletion of KLF4 in the NANOS3-tdTomato reporter ESC line. We prepared the auxin-inducible degron system for ligand-induced targeted protein degradation [21]. Using CRISPR/Cas9 technology, we introduce a tag consisting of the AID degron sequence fused to Venus at the C-terminus of the endogenous KLF4 (figure 2*a*). Subsequently, we utilized the PiggyBac transposon system to deliver codon-optimized transgenes encoding cognate TIR1 to facilitate target degradation upon administration of indole-3-acetic acid (IAA).

We investigated the timing of KLF4 expression during hPGCLC induction using both a Venus reporter and immunostaining with a specific KLF4 antibody (figure 2*b*). Treatment with BMP2 revealed that SOX17 expression precedes the onset of hPGCLC differentiation on day 1, while KLF4 protein expression begins in select hPGCLCs from day 2 onwards and becomes prominent in the majority of SOX17 + hPGCLCs from day 3. The expression pattern observed with the Venus reporter closely resembled the signals detected through KLF4 antibody staining, confirming the successful generation of the reporter line.

We tested changes in KLF4 protein levels upon IAA treatment in the parental control line, which harbours only the TIR integration, and in three distinct KLF4-AID clones. We confirmed that the KLF4-AID fusion protein undergoes degradation in response to IAA treatment mediated by TIR. Upon treatment with IAA, both KLF4 and Venus signals disappeared in SOX17 + hPGCLCs, while no changes in KLF4 expression were observed in the control line (figure 2*c*), confirming the efficient depletion of KLF4 protein.

Next, we investigated the importance of KLF4 for hPGCLC specification by adding IAA at the onset of hPGCLC differentiation (from day 0). We assessed the induction efficiency on day 4 and day 6 by determining the percentage of NANOS3-tdTomato + TNAP + cells by FACS. Compared with hPGCLCs without IAA treatment, the hPGCLC induction efficiency decreased by 30–40% with IAA-induced KLF4 depletion, but not in the control (figure 2*d*). The notable decrease in the efficiency of hPGCLC induction indicates a significant role for KLF4 in hPGCLC differentiation.

### 2.3. KLF4-deficient hPGCLCs show aberrant transcriptome

**Figure 3.**
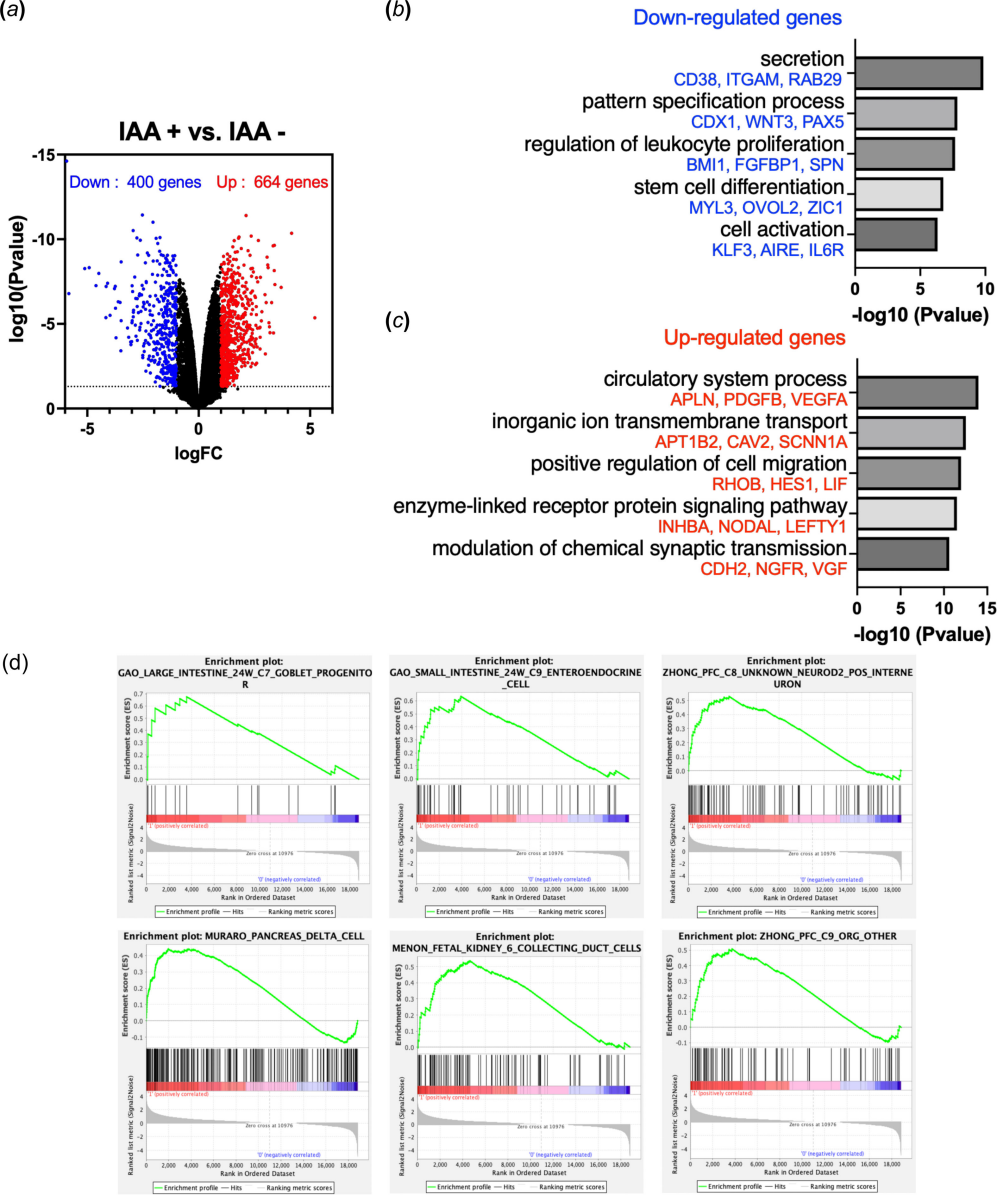
RNA-seq reveals transcriptional changes in KLF4-deficient hPGCLCs. (*a*) Volcano plot showing downregulated (log2FC < −1, *p*_adj_ < 0.05) and upregulated (log2FC > 1, *p*_adj_ < 0.05) genes in hPGCLCs upon KLF4 loss (IAA+ versus IAA−). (*b*) GO analysis on the significantly downregulated genes in KLF4-deficient hPGCLCs. Top non-redundant GO terms are shown as −log10 (*p* value) with example genes included. (*c*) GO analysis on the significantly upregulated genes in KLF4-deficient hPGCLCs. Top non-redundant GO terms are shown as −log10 (*p*-value) with example genes included. (*d*) Enrichment plots of the top six gene set groups enriched for upregulated genes (IAA+ versus IAA−) in KLF4-depleted hPGCLCs were generated using GSEA with the scsig.all.v. 1.0.1.symbols.gmt database.

To determine the transcriptomic changes in KLF4-depleted hPGCLCs, we used KLF4-AID lines. We treated cells with IAA during hPGCLC differentiation and isolated NANOS3-tdTomato + TNAP + hPGCLCs with or without IAA treatment on day 6 using FACS. We then performed bulk RNA-sequencing and compared changes in the expression of protein-coding genes in hPGCLCs from cells treated with IAA versus untreated controls, using three KLF4-AID clones. We observed altered expression patterns of differentially expressed genes (DEGs) in hPGCLCs from IAA-treated cells with a *p*‐value less than 0.05 and log2-fold changes greater than 1. RNA-seq analysis of KLF4-depleted hPGCLCs revealed 664 upregulated and 400 downregulated genes (figure 3*a*).

We performed gene ontology (GO) analysis to explore how gene expression is affected by the loss of KLF4 in hPGCLCs. The downregulated genes were enriched in processes such as secretion, pattern specification and regulation of leukocyte proliferation (figure 3*b*; electronic supplementary material, table S1). Downregulated genes included several PGC regulators like PAX5, OVOL2 and CD38 [22,23]. Conversely, GO analysis of genes upregulated in KLF4-deficient hPGCLCs revealed enrichment in processes related to the circulatory system and synaptic transmission (figure 3*c*; electronic supplementary material, table S1). An abnormal elevation of expression levels was observed for components of signalling pathways such as INHBA and NODAL, which are crucial for endoderm differentiation. We performed gene set enrichment analysis (GSEA) to determine whether the gene sets of specific cell types were enriched for upregulated or downregulated genes in KLF4-depleted hPGCLCs. Using a nominal *p*-value threshold of <0.01, 79 out of 208 gene sets showed enrichment for upregulated genes, whereas no gene sets were enriched for downregulated genes. Among the top six gene set groups, markers of cell types derived from the endoderm lineage—such as goblet and endocrine cells in the intestine—showed significant upregulation, along with markers of some neuronal cell types (figure 3*d*). These findings indicate dysregulation in the expression of key genes required for PGC differentiation and identity in KLF4-depleted hPGCLCs, suggesting that KLF4 modulates gene expression to facilitate PGC development and function.

### 2.4. Characterization of KLF4 binding patterns in hPGCLCs compared with human naive ESCs through cut-and-run analysis

To elucidate the molecular mechanism underlying KLF4-mediated transcriptional regulation, we investigated KLF4 targets by cut-and-run analysis in hPGCLCs. KLF4 is a pluripotency factor that is also expressed in hPGCs, as well as in naive ESCs. Previous studies have shown that the knockdown of KLF4 in naive human ESCs disrupts the formation of human naive ESC colonies [16]. To distinguish the role of KLF4 in PGC differentiation from its role in naive ESCs, we compared and analysed the KLF4 binding patterns in human naive ESCs [19] and hPGCLCs.

**Figure 4.**
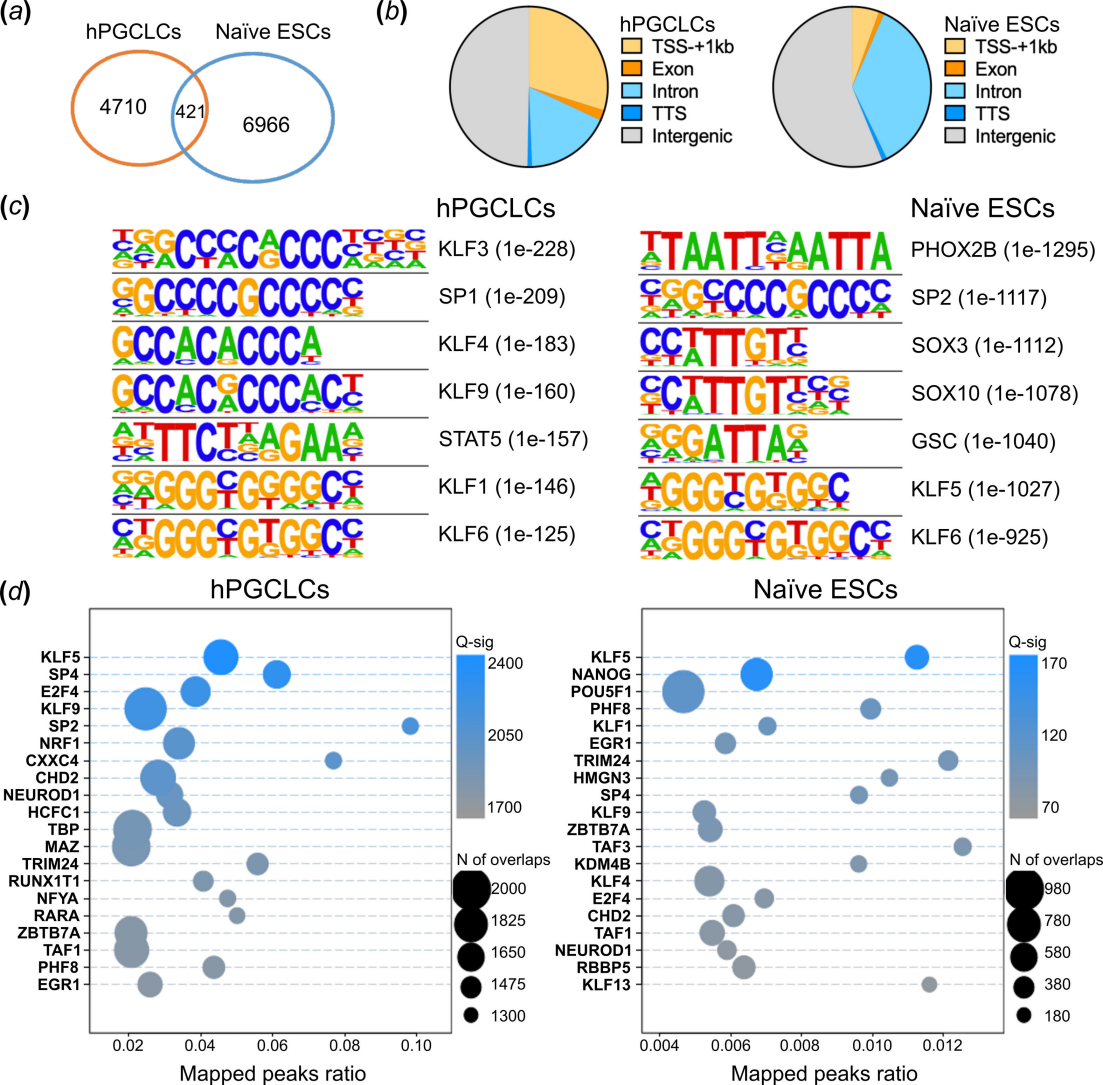
Cut-and-run analysis shows hPGCLC-specific KLF4 binding patterns. (*a*) Venn diagram of KLF4 cut-and-run peaks in hPGCLCs and KLF4 ChIP-seq peaks in naive ESCs overlapping. (*b*)The pie chart presents the distribution of genomic features for KLF4 peaks in hPGCLCs (left panel) and naive ESCs (right panel). (*c*) The topmost commonly known motifs enriched in KLF4 peaks in hPGCLCs (left panel) and naive ESCs (right panel). (*d*) The top scoring ReMap2020 signatures in KLF4 peak regions are shown in hPGCLCs (left panel) and naive ESCs (right panel).

In hPGCLCs, 5131 regions with KLF4 peaks were identified (electronic supplementary material, table S2). Compared with the KLF4 peaks in naive ESCs, only 8.2% overlap was observed (figure 4*a*). The genomic distribution of KLF4 binding sites shows a significant difference between the two cell types. In hPGCLCs, KLF4 predominantly binds within 1 kb of transcription start sites (TSS), with around 30% of peaks spanning annotated promoters. Conversely, KLF4 predominantly binds to distal genomic regions in naive ESCs (figure 4*b*).

The analysis identified the conserved KLF motif as the top-ranking motif in hPGCLCs (figure 4*c*). Additionally, SP1 and STAT5 motifs were co-enriched, suggesting potential cooperation with KLF4. Conversely, motif analysis in naive ESCs revealed co-enrichment of PHOX2B, SOXs and GSC transcription factors. Similarly, ReMap enrichment analysis also revealed distinct co-enrichment patterns of factors on KLF4 binding sites in hPGCLCs compared with naive ESCs. In naive ESCs, KLF4 potentially co-localized with other pluripotency factors, such as NANOG and OCT4 (figure 4*d*), but in hPGCLCs, this co-localization was not observed; instead, SPs and E2F4 factors were identified (see §3).

### 2.5. Integration of cut-and-run and RNA-seq datasets identifies potential direct targets of KLF4-mediated transcriptional regulation in hPGCLCs

**Figure 5.**
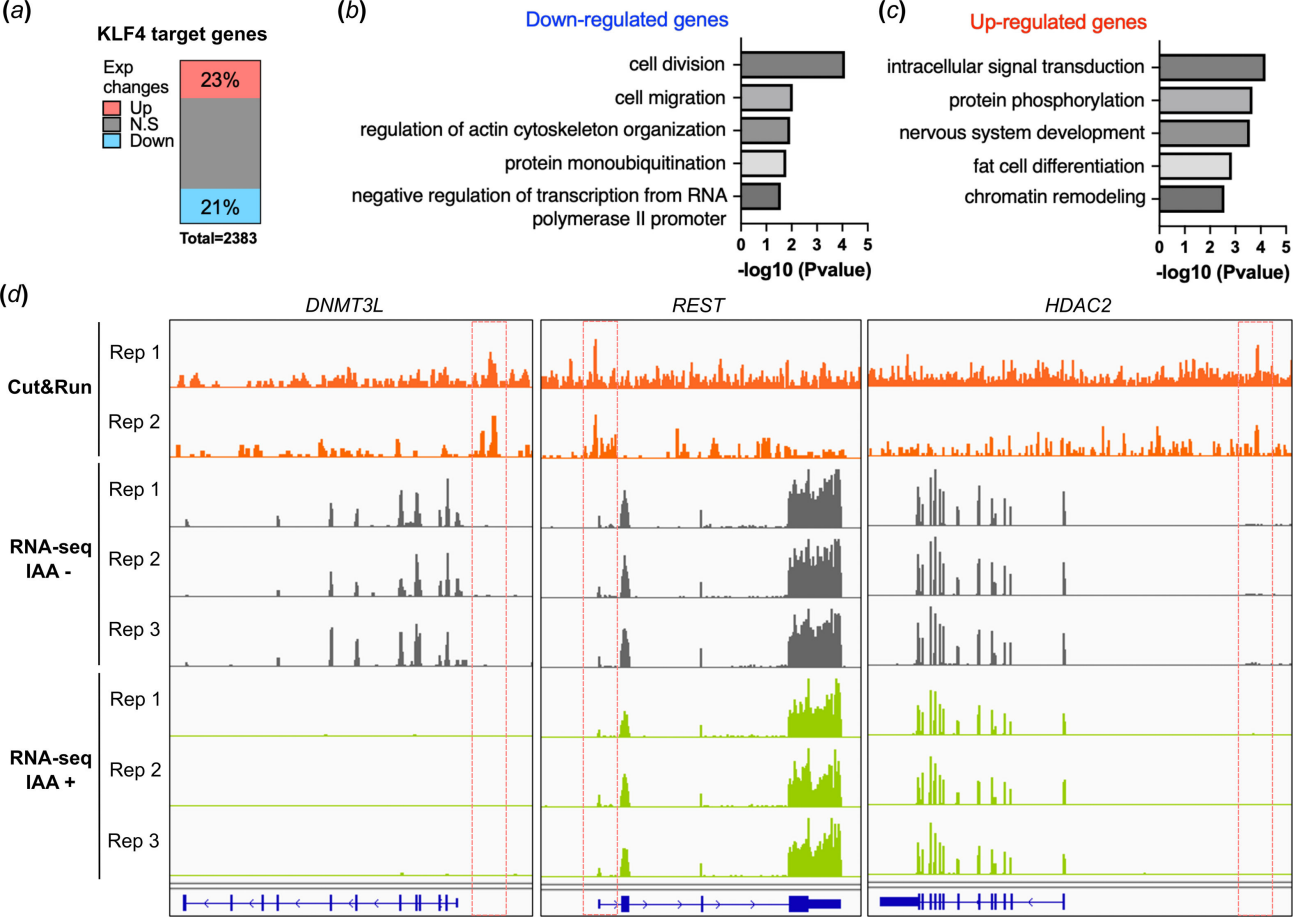
KLF4-targeted gene expression changes in KLF4-deficient hPGCLCs. (*a*) The bar plot represents the percentage of overlap between KLF4 target genes showing significant changes in expression upon IAA treatment in KLF4-AID hPGCLCs. Proportions of upregulated and downregulated genes (*p*‐value < 0.05) in KLF4-deficient hPGCLCs are indicated. (*b*) Gene ontology (GO) biological process terms significantly enriched for KLF4-targeted and downregulated genes are shown. (*c*) GO terms significantly enriched for KLF4-targeted and upregulated genes are shown. (*d*) Visualization for KLF4 cut-and-run peaks, bulk RNA-seq at *DNMT3L*, *REST* and *HDAC2* genomic loci.

We investigated the impact of KLF4 depletion on the expression of KLF4 target genes in hPGCLCs. We identified 2383 protein-coding genes that are closely located to peaks and are considered KLF4 target genes. Upon treatment with IAA in the KLF4-AID line, we observed significant changes in gene expression in hPGCLCs. Specifically, 21% of the target genes exhibited decreased expression, while 23% of the genes showed increased expression as a result of KLF4 depletion (figure 5*a*).

When analysing the GO terms, the expression of different gene groups (increased or decreased) was observed. The downregulated genes were enriched in GO terms related to various cellular processes, including ‘cell division’, ‘cell migration’, ‘actin cytoskeleton organization’ and ‘negative regulation of transcription from RNA polymerase promoter’ (figure 5*b*). Conversely, the upregulated genes showed enrichment in processes such as ‘intracellular signal transduction’, ‘nervous system development’, ‘fat cell differentiation’ and ‘chromatin remodelling’ (figure 5*c*). It appears that KLF4 directly inhibits neuronal or fat cell differentiation in hPGCs. This inhibition is relieved upon KLF4 depletion, leading to the upregulation of somatic genes. The suppression of genes that induce differentiation of various somatic cell types during PGC specification plays a crucial role in maintaining PGC identity, and KLF4 seems to contribute to this regulatory function.

KLF4 also plays a role in the cell cycle and migration in various cell types, notably in PGCs [24]. Additionally, KLF4 regulates several transcriptional repressors, including *DNMT3L*, *REST* and *HDAC2*. KLF4 binding sites were observed in the promoter regions of these genes or upstream of *HDAC2*, and a decrease in the expression of these genes was observed in KLF4-depleted hPGCLCs (figure 5*d*). The reduced expression of these repressors may subsequently cause the loss of repression of somatic genes in hPGCs or of various gene expression regulators.

## 3. Discussion

Depletion of KLF4 using an acute protein degradation strategy affected hPGCLC specification efficiency, resulting in a 30–40% decrease, possibly due to transcriptional abnormalities. Loss of KLF4 in hPGCLCs suppresses the expression of genes involved in pattern specification processes and stem cell differentiation, including PAX5 and OVOL2. PAX5 is indeed recognized as one of the regulators of hPGC development, operating upstream of OCT4 and PRDM1. The three-factor network functions to activate germline programmes while simultaneously repressing somatic programmes [22]. Ovol2 plays a role in repressing genes associated with epithelial-to-mesenchymal transition (EMT) and induces the expression of genes related to PGC specification [23]. Several factors involved in PGC specification and maintenance are suppressed in KLF4-depleted hPGCLCs. As a result, CD38, which is an early marker for PGCs, exhibits reduced expression in hPGCLCs when KLF4 is depleted.

KLF4 can act as both a transcriptional activator and a repressor. During the reprogramming of somatic cells into induced pluripotent stem cells (iPSCs), KLF4 initially suppresses differentiation markers and later promotes the expression of pluripotency genes [25]. Analysis of KLF4-depleted hPGCLCs indicates its involvement in maintaining the early human germ cell programme by suppressing alternative fates. Interestingly, key components of the NODAL/activin signalling pathway, NODAL and INHBA (activin A receptor genes), are derepressed in KLF4-depleted hPGCLCs. These factors activate SMAD2/4, which is a core regulatory pathway for definitive endoderm differentiation. For PGC specification, NODAL/activin signalling is required, but these genes are repressed afterwards. KLF4 knock-down in mouse ESCs leads to differentiation towards visceral and definitive endoderm [26]. In KLF4-depleted hPGCLCs, there is an abnormal upregulation of genes related to somatic differentiation, including endodermal and neuronal genes. KLF4 directly regulates neuronal genes in hPGCLCs, similar to its role in ESCs [27].

KLF4 binding directly influences the function of many epigenetic repressors. For instance, HDAC2, an interacting partner of PRDM1, and REST, a key repressor that forms complexes with HDACs, are regulated by KLF4. Furthermore, KLF4 depletion leads to reduced expression of *DNMT3L* in KLF4-depleted hPGCLCs. DNMT3L collaborates with DNMT3A in PGCs to methylate imprinted regions and transposable elements [28,29]. In summary, KLF4 seems to play a role in shaping the epigenome of PGCs.

We observed different binding patterns of KLF4 in hPGCLCs and naive ESCs, indicating cell type-specific regulatory roles for KLF4. In naive ESCs, KLF4 predominantly binds to intergenic and intronic regions with SOX/NANOG/POU5F1 enrichment at the binding sites. By contrast, in hPGCLCs, KLF4 frequently binds to the promoter regions and shows co-enrichment of SPs, E2F4 and STAT5, which are essential for cell cycle regulation [30,31]. This suggests that KLF4 may play a role in regulating the proliferation of hPGCLCs and is involved in pluripotency networks in naive ESCs.

Genes downregulated in the absence of KLF4 primarily include those involved in regulating cell division, migration and transcription. KLF4 was initially identified as a growth arrest factor due to its association with cell cycle regulation [32,33]. The number of PGCs is dynamically and tightly regulated during development to ensure that an adequate and precise number of PGCs are available to generate gametes, ensuring fertility and germline integrity. The regulation of cell numbers involves controlling the cell cycle and apoptosis; KLF4 is expected to play a role in this process [24]. In mice, PGC proliferation happens more quickly over a few days, but in humans, it must be carefully regulated over several weeks [2,34]. KLF4, present in humans but not mouse PGCs, is likely to have a more significant role in this regulation in human PGCs. Genes linked to cell migration are direct targets of KLF4 after PGC specification. This indicates that KLF4 might also play a role in cell migration, as seen in the analysis of KLF4-depleted cells. The STAT factors, which could potentially collaborate with KLF4, are known to be involved in PGC migration [35].

KLF4 has a context-dependent role in PGCs and naive pluripotency, which is consistent with our observations. We discovered that KLF4 regulates the expression of multiple genes essential for PGC development, acting both as a transcriptional activator and a repressor. Our investigation of KLF4 has broader implications for understanding human germline biology and related disorders.

## 4. Methods

### 4.1. Cell culture

NANOS3–tdTomato reporter for WIS2 male ESCs were established previously [4]. 4iESCs were maintained on irradiated mouse embryonic fibroblasts (MEFs) (purchased from MTI-GlobalStem) in knockout DMEM (Thermo Fisher Scientific) supplemented with 20% knockout serum replacement, 0.1 mM non-essential amino acids, 0.1 mM 2-mercaptoethanol, 100 U ml^−1^ penicillin, 0.1 mg ml^−1^ streptomycin, 2 mM l-glutamine, 20 ng ml^−1^ human LIF (Stem Cell Institute, University of Cambridge (SCI)), 8 ng ml^−1^ bFGF (SCI), 1 ng ml^−1^ TGFβ (Peprotech), 3 μM GSK3i (CHIR99021, Miltenyi Biotec), 1 μM ERKi (PD0325901, Miltenyi Biotec), 5 μM p38i (SB203580, TOCRIS Bioscience) and 5 μM JNKi (SP600125, TOCRIS Bioscience) [3]. Cells were passaged every 3−5 days using TrypLE Express (Thermo Fisher Scientific). Before seeding 4i ESCs on MEFs, 10 μM of ROCKi (Y-27632, TOCRIS Bioscience) was added to the medium. To induce PGCLCs, 4i ESCs were trypsinized into single cells and seeded into Corning Costar Ultra-Low attachment multiwell 96-well plates (Sigma) at 4000 cells per well in hPGCLC induction medium composed of aRB27 medium supplemented with 500 ng ml^−1^ BMP2 (SCI),10 ng ml^−1^ human LIF (Department of Biochemistry), 100 ng ml^−1^ SCF (R&D Systems), 50 ng ml^−1^ EGF (R&D Systems) and 10 μM ROCKi. Cells were cultured as floating aggregates for 4−6 days. For AID experiments, IAA was added to the cell culture medium at a final concentration of 100 μM.

### 4.2. Plasmid construction and cell line establishment

To generate KLF4-AID-mVenus reporter lines, knock-in donor plasmids were constructed by Gibson assembly. The plasmids consisted of a KLF4 5′ homology arm amplified by PCR from genomic DNA, an AID-mVenus sequence in-frame with the C-terminus of KLF4, a selection marker driven by the PGK promoter and flanked by Rox sites (recognition sites for Dre recombinase), and a KLF4 3′ homology arm amplified by PCR from genomic DNA. Two versions of the plasmid were constructed, one with a NeoR selection marker and one with a Puro∆TK selection marker. The plasmid backbone additionally contained an MC1-DTA cassette to select against random integration. Additionally, a CRISPR targeting plasmid was constructed by ligating sgRNA oligos (caccGTGTGGGTCATATCCACTGTC/aaacGACAGTGGATATGACCCACAC) into the pX330 Cas9/sgRNA expression vector (Addgene no. 42230).

The targeting and donor plasmids were lipofected into WIS2 NANOS3–tdTomato hESCs. For each lipofection, 200 000 cells were used in 100 µl OptiMEM (Gibco) with 2 µg targeting plasmid, 2 µg donor plasmid and 4 µl lipofectamine stem (Thermo Fisher). Cells were seeded on DR4 MEFs in 4i medium + 10 µM Y27632. In order to obtain bi-allelic edited lines, cells were first lipofected with the targeting plasmid and NeoR donor plasmid and selected with G418 (200 µg ml^−1^) starting two days after lipofection. After the selection was complete, cells were lipofected again with the targeting plasmid and Puro∆TK donor plasmid and selected with puromycin (0.4 µg ml^−1^) starting two days after lipofection. Single colonies were picked, and editing was confirmed by PCR and Sanger sequencing. To remove the selection markers, cells were lipofected with a Dre expression plasmid. Selection with FIAU (200 nM) starting two days after lipofection was performed to eliminate any cells retaining puro∆TK expression.

To introduce the TIR1 ubiquitin ligase, 200 000 cells were lipofected as described above, with 1 µg of a PiggyBac transposase expression plasmid (Systems Bioscience) and 1 µg of a transposon plasmid expressing TIR1-IRES-HygroR driven by the constitutive EF1a promoter. Cells were selected with hygromycin (50 µg ml^−1^) starting two days after lipofection.

### 4.3. Flow cytometry analysis

hPGCLC induction was performed as described above. To dissociate cells, aggregates were collected, washed with PBS and digested with 0.25% trypsin/EDTA (5 μl per aggregate) for 10 min at 37°C with gentle shaking (600 rpm). For day 6 and older aggregates, dissociation was completed by passing the suspension multiple times through a 27-gauge needle. Trypsin was quenched with two volumes of ice-cold sorting medium (3% FBS in PBS) and the cells were pelleted (300*g*, 2 min). Next, the cells were re-suspended in sorting medium (5 μl per EB) containing AF647 conjugated mouse anti-human TNAP IgG (BD Biosciences 561500; RRID: AB_10717125) (1 μl per 12 EBs) and incubated at 4°C for 30 min. The antibody solution was diluted with two volumes of sorting medium, and the cells were pelleted (300*g*, 2 min) and resuspended in 500 μl sorting medium plus DAPI (0.1 μg ml^−1^). The suspension was filtered with a 50 μm strainer and analysed on a BD LSRFortessa flow cytometer. A Sony 800Z cell sorter was employed to isolate DAPI-negative cells, followed by sorting TNAP + NANOS3-tdTomato + hPGCLCs.

### 4.4. Generation of RNA-seq libraries

hPGCLCs (20 000–76 000 per sample) were FACS-sorted directly into 50 μl RNA extraction buffer (Arcturus PicoPure), which was frozen at −80°C for subsequent use. RNA was extracted with the Arcturus PicoPure kit following the manufacturer’s instructions. Library preparation was performed using the NEBNext Ultra II Directional RNA Library Prep Kit (NEB #E7760, polyA mRNA workflow) following the manufacturer’s protocol. Sequencing was performed on an Illumina NextSeq 500.

### 4.5. Cut-and-run and library preparation

Cut-and-run for KLF4 was performed as described in [36]. Briefly, 50 000 purified NANOS3 + TNAP + hPGCLCs (day 4) were washed and bound to activated 10 μl concanavalin A-coated magnetic beads. The cell-bound beads were incubated with wash buffer containing 0.1% digitonin and 0.6 μg of KLF4 antibody (ab215036, Abcam) for 2 h at 4°C on a rotator. After two washes in digitonin-wash buffer, the cell-bound beads were resuspended in protein A/G-MNase fusion protein at 70 ng ml^−1^ in digitonin-wash buffer and incubated for 1 h at 4°C on a rotator. After two washes in digitonin-wash buffer, the cell-bound beads were resuspended in ice-cold calcium incubation buffer. After 15 min, 2× stop buffer was added. The cell-bound beads were incubated at 37°C for 30 min; the liquid was removed to a fresh tube and DNA was extracted with phenol–chloroform extraction. The buffer compositions are as follows: wash buffer containing 20 mM HEPES (pH 7.5), 150 mM NaCl, 0.5 mM spermidine and protease inhibitor; calcium incubation buffer containing 3.5 mM HEPES (pH 7.5), 10 mM CaCl2 and 0.1% digitonin; and 2× stop buffer containing 340 mM NaCl, 20 mM EDTA, 4 mM egtazic acid, 0.1% digitonin, RNase A 100 μl ml^−1^ and glycogen 50 μg ml^−1^.

Sequencing libraries were prepared using the NEBNext Ultra II DNA Library Prep Kit (NEB, E7645S) from Illumina according to the manufacturer’s instructions. PCR enrichment of adaptor-ligated DNA was conducted with the KAPA HiFi Real-Time PCR Library Amplification Kit (Roche, KK2702) as per the manufacturer’s guidelines (13–14 PCR cycles). SPRIselect beads (Beckman Coulter, B23317) were utilized for PCR product clean-up and size selection. Libraries were sequenced for 150 cycles in paired-end mode on the NovaSeq platform.

### 4.6. Data processing for bulk RNA-seq

The low-quality reads and adaptor sequences were removed by Trim Galore. The pre-processed reads were mapped to the human reference genome (GENCODE, GRCh38.p13) using STAR with parameters—outFilterMultimapNmax 1—outFilterMatchNmin 35. The raw read counts per gene were extracted by the—quantMode GeneCounts option. Differential gene expression analysis was performed with the glm method of the edgeR package for protein-coding genes. The HOMER functions makeTagDirectory and makeUCSCfile were used to create bedGraph files. GO analysis was performed with metascape (http://metascape.org) and DAVID Knowledgebase (https://david.ncifcrf.gov). Bulk-RNA seq results used in figure 1*a* were downloaded from GSE60138 and NCBI SRA: SRP057098. Marker protein-coding genes, 142 for migratory, 288 for mitotic and 937 for mitotic arrest male PGCs, were used on the basis of published markers identified from single-cell RNA-seq data [20,37]. GSEA (v. 4.3.3) was performed using the scsig.all.v. 1.0.1.symbols.gmt database [38].

### 4.7. Data processing for cut-and-run

The leeHom package program with —ancient dna option was used to trim the reads. The pre-processed reads were mapped to the human reference genome (GENCODE, GRCh38.p13) using Bowtie2 with options very-sensitive–no-mixed–no-discordant-q–phred33-I 10-X 700. For MACS2 peak calling, parameters used were macs2 callpeak–keep-dup all–bdg. A total of 5131 peaks that are in common between the replicates were used for further analysis. Peaks were annotated to their nearest genes using Homer annotatePeaks.pl function. HOMER findMotifsGenome.pl function was used to analyse the enriched TF motifs over peaks. To calculate statistical enrichments of TFs on the peaks based on the ReMap database, we used ReMapEnrich Shiny 1.4. KLF4 peak regions in human naive ESCs were downloaded from GSM3738360, and peaks with a score over 100 were used.

### 4.8. Immunofluorescence

hPGCLC-containing embryoid bodies (EBs) were fixed using 4% paraformaldehyde (PFA) in PBS at 4°C for 1−2 h. Human embryonic genital ridges from individual embryos were dissected in PBS and separated from surrounding mesonephric tissues. The genital ridges were fixed in 2.5% PFA in PBS overnight at 4°C. After two washes in PBS, the EBs or genital ridges were incubated in 10% sucrose solution in PBS for 1 day and then 20% sucrose in PBS for 1 h at 4°C. The EBs or genital ridges were embedded in OCT compound (CellPath), snap frozen on dry ice and stored at −80°C until cryosectioning. Cryosections were made using a Leica Microsystems cryostat.

The sections were air-dried at RT for 1 h, washed in PBS three times and permeabilized in 0.1% Triton X-100 in PBS for 30 min at RT. The sections were incubated with the blocking solution (0.1% Triton X-100, 5% normal donkey serum (Stratech)) for 30 min at RT. The sections were then incubated with primary antibodies diluted in the blocking buffer overnight at 4°C. The following day they were washed three times with the wash buffer (0.1% Triton X-100 in PBS) and incubated with Alexa fluorophore-conjugated secondary antibodies (Invitrogen, used in 1:500 dilutions in the 0.1% Triton X-100 in PBS with 1 μg ml^−1^ DAPI) for 1 h at RT in the dark. The sections were washed in the wash buffer three times and in PBS once and mounted with Vectashield (Vector Laboratories). The images were acquired using Leica SP5 or SP8 confocal microscope and analysed using Fiji software. Primary antibodies (anti-POU5F1, mouse, monoclonal, BD Biosciences, cat. no. 611203; anti-SOX17, goat, polyclonal, R&D Systems, cat. no. AF1924; Recombinant Anti-KLF4, rabbit, monoclonal, Abcam, cat. no. ab215036; Anti-KLF4, goat, polyclonal, R&D Systems, cat. no. AF3640) and fluorescent-conjugated secondary antibodies (TRITC, Alexa fluorophore 488, 555, 568 and/or 647; eBioscience and Thermo Fisher Scientific) were used.

## Data Availability

The sequencing data have been deposited in the Gene Expression Omnibus under GSE271818. Supplementary material is available online [39].
